# Microscopic—II

**Published:** 1880-07

**Authors:** S. O. Gleason


### MICROSCOPIC---II.

S. O. GLEASON, M. D.

Let any one who has a microscope, take all the sections of a common jointed fishing rod, except the slender tip, and get a small tin dipper, say three inches in diameter, a little flaring with a short hollow handle; insert the rod into the same and put on all the other sections and one can thus reach out into boggy and marshy places and get water, a little in one place and then some from another, till all is obtained that is needed for a great deal of work.

Take water thus gathered and put it in dishes two inches deep, keep the same where there is plenty of light but do not expose to the hot sun, as all forms of life will soon die under too much sun heat.

In nearly all wet places and even in damp earth, ponds, and little streams can be found plenty of diatoms, desmids, infusoria, rotafers, rhizopods, amœba, etc., etc. We will give a brief description of the rhizopod. Its name is made up of two greek words, rhiza—root, pous—foot.

15—In the next verse of the ballad, without is spelled w-i-t-h-o-w-g-h-t-e-n, strife, s-t-r-y-f-f-e, and earl, y-e-r-l-l-e.

16—I wanted to say that, "they had bitten off more than they could chew," it does so cover the whole ground.

We then have root-footed animals, many of them are scarcely visible to the naked eye, while others can be seen as little flakes of jelly-like material.

Though small in size they are so multitudinous in numbers that they make huge masses of life in all the waters of the world.

One can hardly fail to find them in marsh, pool, ditch, pond, lake and sea. Even in the very depths of the ocean they find a home.

They are thought to be the earliest manifestations of life on the earth. So, if one believes in the theory of evolution, they represent the first beginning of all forms of animal life, even the human race. Many of these minute forms have shells and make up a large part of some kinds of rocks.

The more simple form of rhizopod has no shell, no firm covering of any kind.

They seem to be made up of a soft substance like jelly, viscid, like gum-arabic water, having power to expand and contract, thus to change shape and execute a variety of movements.

The main substance of this form of life, is called protoplasm (greek, *protos*, first, plasso, I mould,) this material seems to be the primitive matter from which all organized bodies are made.

These small animals have no mouth, no stomach, no intestines, no mouths. Yet they move about with freedom, swallow food, appropriate it, and reject what they cannot use. They increase in size and reproduce their kind. Many wonderful forms of beauty are evolved and many marvelous attributes made manifest by these tiny forms of life.

To move, the rhizopod extends or protrudes portions of its substance, which act as temporary organs of locomotion. These parts are withdrawn and make part of its body again. So when in motion, it is constantly assuming new forms like the fabled Proteus. These extensions are called pseudopods, or false feet. They may be either thread-like, extremely delicate, or broader and more like fingers. Sometimes they branch and assume a *root-like* appearance, hence the name rhizopod.

Thus, the simplest form of rhizopod, is but a little mass of naked unprotected protoplasm. The greater number of these animals are protected by some kind of a skeleton frame work, made of silex, or a shell of chitinoid membrane, or of limestone. The hard part is mostly *inside*, but at times it is *outside*, making a shell of siliceous matter. We find a great variety of forms, shapes and some of them exhibit very remarkable beauty and evident design in their construction.

This little sketch has been written to encourage young people who have leisure to use the microscope, and thus extend their researches into this charming field of investigation.

